# Molecular Diagnostic and Pathogenesis of Hereditary Hemochromatosis

**DOI:** 10.3390/ijms13021497

**Published:** 2012-02-01

**Authors:** Paulo C. J. L. Santos, Jose E. Krieger, Alexandre C. Pereira

**Affiliations:** Laboratory of Genetics and Molecular Cardiology, Heart Institute (InCor), University of Sao Paulo Medical School, SP, 05403-000, Brazil; E-Mails: krieger@incor.usp.br (J.E.K.); acplbmpereira@gmail.com (A.C.P.)

**Keywords:** hemochromatosis, primary iron overload, *HFE*, high-resolution melting, *HJV*, molecular diagnostic

## Abstract

Hereditary hemochromatosis (HH) is an autosomal recessive disorder characterized by enhanced intestinal absorption of dietary iron. Without therapeutic intervention, iron overload leads to multiple organ damage such as liver cirrhosis, cardiomyopathy, diabetes, arthritis, hypogonadism and skin pigmentation. Most HH patients carry *HFE* mutant genotypes: homozygosity for p.Cys282Tyr or p.Cys282Tyr/p.His63Asp compound heterozygosity. In addition to *HFE* gene, mutations in the genes that encode hemojuvelin (*HJV*), hepcidin (*HAMP*), transferrin receptor 2 (*TFR2*) and ferroportin (*SLC40A1*) have been associated with regulation of iron homeostasis and development of HH. The aim of this review was to identify the main gene mutations involved in the pathogenesis of type 1, 2, 3 and 4 HH and their genetic testing indication. *HFE* testing for the two main mutations (p.Cys282Tyr and p.His63Asp) should be performed in all patients with primary iron overload and unexplained increased transferrin saturation and/or serum ferritin values. The evaluation of the *HJV* p.Gly320Val mutation must be the molecular test of choice in suspected patients with juvenile hemochromatosis with less than 30 years and cardiac or endocrine manifestations. In conclusion, HH is an example that genetic testing can, in addition to performing the differential diagnostic with secondary iron overload, lead to more adequate and faster treatment.

## 1. Introduction

Hereditary hemochromatosis (HH) is an autosomal recessive disorder characterized by enhanced intestinal absorption of dietary iron. Without therapeutic intervention, iron overload leads to multiple organ damage such as liver cirrhosis, cardiomyopathy, diabetes, arthritis, hypogonadism and skin pigmentation. However, iron can be efficiently and safely removed by therapeutic phlebotomy, which is initiated by withdrawing blood at a rate of 500 mL per week until serum ferritin reaches <50 μg/L. The oral iron chelator deferasirox is not registered for genetic iron overload, since conventional phlebotomies have much lower side effects. But recent studies reported that oral chelator could be used in exceptional cases [1–5].

The major mutation that has been associated with disease is the p.Cys282Tyr in the *HFE* gene that occurs in approximately 80% of HH cases. In addition, a high proportion of the remaining patients are compound heterozygous for the *HFE* p.Cys282Tyr and the common *HFE* p.His63Asp alteration [6–8]. In Northern European populations, the *HFE* p.Cys282Tyr homozygous genotype is particularly common (1 in 200–300 healthy subjects) and the *HFE* 282Tyr allele frequency is high (5.1 to 8.2%) [9]. In contrast, in countries with racial/ethnic heterogeneity from South America, Asia and Africa a lower prevalence of HH have been observed, and an increased number of patients with primary iron overload do not carry the p.Cys282Tyr/p.Cys282Tyr or p.Cys282Tyr/p.His63Asp genotypes [10,11] (for example, a minor allele frequency of p.Cys282Tyr allele of 2.3% is observed in Brazilian blood donors) [12,13].

In addition to the *HFE* gene, mutations in the genes that encode hemojuvelin (*HJV*), hepcidin (*HAMP*), transferrin receptor 2 (*TFR2*) and ferroportin (*SLC40A1*) have been associated with regulation of iron homeostasis and development of HH [9,14,15].

Early diagnostic and initiation of iron-depletion therapy ensure life quality and increase survival times of HH patients. In this scenario, genetic testing applied to HH can, in addition to performing the differential diagnostic with secondary iron overload, lead to more adequate and faster treatment. Thus, the pivotal aim of this review was to identify the main gene mutations involved in the pathogenesis of the type 1, 2, 3 and 4 HH and their genetic testing indications.

## 2. HH Types, Related Genes and Their Main Mutations

According to OMIM (Online Mendelian Inheritance in Man, www.ncbi.nlm.nih.gov/omin) 5 types of HH have been identified on the basis of clinical, biochemical, and genetic characteristics (Table 1). The classic hemochromatosis is most often caused by a mutation in a gene designated *HFE* on chromosome 6p21.3. Nonetheless, in minor frequency, there are 4 additional disorders of primary iron overload: juvenile hemochromatosis (JH) or type 2 hemochromatosis, which is divided into 2 forms: type 2A JH, caused by mutations in the *HJV* gene on chromosome 1q21, and type 2B JH, caused by mutations in the *HAMP* gene on chromosome 19q13. HH types 3 and 4 are caused by mutations in the *TFR2* and *SLC40A1* genes on chromosomes 7q22 and 2q32, respectively (Table 1) [16–19].

### 2.1. HFE

*HFE* related-HH (OMIM 235200), classified as type 1, is the most frequent form of the disease and the most common autosomal recessive disorder in Northern European populations. HH is characterized by enhanced intestinal absorption of iron leading to multiple organ damage, such as cirrhosis, hepatoma, diabetes mellitus, arthritis, cardiomyopathy, and hypogonadism [20–23].

*HFE* gene (613609), constituted by 6 exons, encodes a membrane protein that is similar to major histocompatibility class I-like proteins, called HFE.

Most HH patients carry homozygosity for p.Cys282Tyr or p.Cys282Tyr/p.His63Asp compound heterozygous genotypes. Besides the missense mutation at position 282, where cysteine is replaced by tyrosine (p.Cys282Tyr, c.845G>A, rs1800562) and the common substitution of histidine for aspartic acid at position 63 (p.His63Asp, c.187C>G, rs1799945), a third mutation is also commonly assessed: the substitution of cysteine for serine at amino acid position 65 (p.Ser65Cys, c.193A>T, rs1800730). However, recent reports have suggested that rare *HFE* variants, such as p.Gly43Ala, p.Leu46Trp, p.Val53Met, p.Gly93Arg, p.Ile105Thr, p.Gln127His, p.Asp129Asn, p.Glu168Gln, p.Glu168del, p.Leu183Pro, p.Glu277Lys, p.Gln283Pro, p.Val284Met, p.Arg330Met, and a deletion in the 6p chromosome region containing *HFE* could also be linked to HH thus contributing to genetic and phenotypic heterogeneity of the disease [6,7,9,15,24,25].

**Figure 1:**
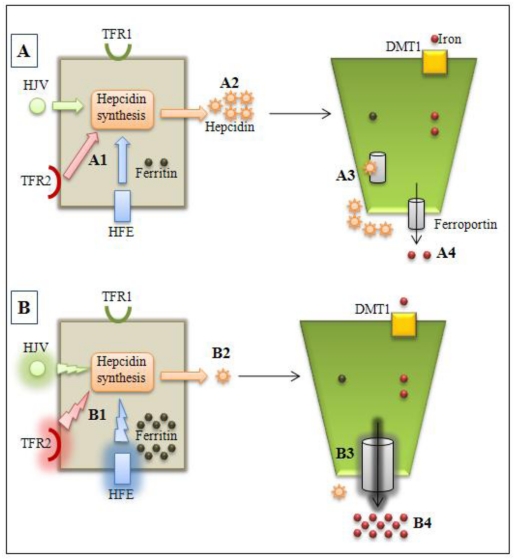
Normal (**A**) and hemochromatosis (**B**) conditions. A1: HFE, HJV, and TFR2 modulates hepcidin synthesis by hepatocytes; A2: normal hepcidin levels; A3: hepcidinferroportin interaction with internalization and ferroportin degradation in enterocytes; A4: normal iron absorption. B1: *HFE* or *HJV* or *TFR2* gene mutations alter hepcidin synthesis modulation; B2: lower hepcidin levels; B3: decreased hepcidin-ferroportin interaction and increased ferroportin activity; B4: iron overload observed in types 1, 2 and 3 hemochromatosis. TFR2: transferrin receptor 2; TFR1: transferrin receptor 1, HFE: HFE protein; HJV: hemojuvelin.

The first proposed pathogenic mechanism for explaining HH was the disruption of a disulfide bond in HFE that is critical for its binding to β2 microglobulin. This complex interacts with transferrin receptor 1, decreasing the affinity with transferrin and consequently modulating iron absorption in enterocytes [26]. However, in recent years, evidences indicating HFE protein as a hepcidin modulator have emerged. The functional loss of HFE in mice and humans has been shown to reduce hepcidin synthesis [27–29] and that HFE loss seems to be associated with blunted signaling responses to BMP6 (bone morphogenetic protein 6), a key regulator of hepcidin, *in vitro* and *in vivo* [30,31]. Indeed, *HFE* related-HH has been associated with reduced hepcidin levels (Figure 1) [24,28,29].

### 2.2. HJV and HAMP

Juvenile hemochromatosis (JH), also classified as type 2, is a rare autosomal recessive disorder of iron overload that leads to organ damage before the age of 30, and usually causes cardiomyopathy, hypogonadotrophic hypogonadism, liver damages and endocrine dysfunctions. Types 2A (OMIM 602390) and 2B (OMIM 613313) are caused by mutations in *HJV* and *HAMP* genes, respectively [32,33].

*HJV* (608374) gene, constituted by 4 exons, was identified in 2004 and encodes a protein called hemojuvelin [32]. Patients with type 2A JH and knockout mice models demonstrate low hepcidin levels implying that hemojuvelin is involved in the hepcidin synthesis [34]. *HAMP* gene (606464), constituted by 3 exons, encodes hepcidin, a peptide known as iron hormone. Hepcidin is produced by hepatocytes and it plays a role in iron absorption related to ferroportin degradation of the enterocytes [33,35].

Several *HJV* mutations have been found in patients: p.Arg54del, p.Cys80Arg, p.Ser85Pro, p.Gly99Arg, p.Gly99Val, p.Leu101Pro, p.Gly116del, p.Cys119Phe, p.Ile222Asn, p.Arg131fs, p.Asp149fs, p.Leu165del, p.Ala168Asp, p.Phe170Ser, p.Asp172Glu, p.Arg176Cys, p.Trp191Cys, p.Asn196Lys, p.Ser205Arg, p.Ile222Asn, p.Lys234del, p.Asp249His, p.Gly250Val, p.Asn269fs, p.Ile281Thr, p.Arg288Trp, p.Cys321Trp, p.Cys321del, p.Arg326del, p.Ser328fs, p.Cys361fs, and p.Arg385del. However, the *HJV* p.Gly320Val is the most frequent mutation and has been reported in JH patients in several populations around the world [22,32,36–39]. In contrast, mutations in *HAMP* are a very rare cause of JH: p.Met31fs, p.Met50fs, p.Arg56del, p.Arg59Gly, p.Cys70Arg, p.Gly71Asp, and p.Cys78Thr [22,33,40,41]. In addition, some studies support the concept that digenic inheritance of *HFE* and *HJV* or of *HFE* and *HAMP* mutations can lead to iron overload or may aggravate the phenotype [37,39,41–44].

For both type 2A and 2B JH, it is well established that the cause of iron overload may be explained by decreases in the synthesis and, consequently depressed hepcidin levels (Figure 1) [34,45]. Cell-surface expression of hemojuvelin was associated with increased expression of hepcidin; likewise, loss of hemojuvelin expression, as in juvenile hemochromatosis, was associated with reduced hepcidin expression [22,46].

HJV seems to play a role in iron absorption and release from cells and has anti-inflammatory properties [47]. An important study revealed that HJV acts as a BMP co-receptor and signals via the SMAD pathway to regulate hepcidin expression [46,48]. A BMP6 dependent signaling pathway has been shown to play a key role in regulation of hepcidin expression [24]. BMPs bind to type I and type II serine threonine kinase receptors, which phosphorylate specific intracellular SMAD proteins (SMAD1,5,8). Phosphorylated SMAD1,5,8 (P-SMAD1,5,8) binds to the common mediator SMAD4, and the SMAD complex translocates to the nucleus to affect transcription of target genes *HAMP* (encoding hepcidin) is transcriptionally up-regulated by BMPs [46,49–52]. Impaired hepatic signaling through mutations in genes encoding either the ligand BMP6, the BMP coreceptor hemojuvelin or Smad4 leads to low hepcidin levels and iron overload in mice. Collectively, these data show that BMP-SMAD signaling is an important regulatory pathway for hepcidin expression and thus iron metabolism [53–56].

### 2.3. TFR2

Type 3 HH (OMIM 604250) is an autosomal recessive disease caused by mutations in *TFR2* gene and iron overload is similar to *HFE* related-HH phenotype. *TFR2* gene (604720), constituted by 18 exons, encodes transferrin receptor 2 protein (TFR2). TFR2 is involved with uptake of transferrin bound iron by hepatocytes and it is also involved in the hepcidin synthesis [57–60]. One possibility is that it operates in the pathway discussed above for HFE (or in a parallel pathway of its own) facilitating the BMP/SMAD signaling that activates hepcidin expression. Another possibility is that TFR2, which is also able to interact with HFE, forms an iron-sensing complex that modulates hepcidin expression in response to blood levels of diferric transferrin [24,61–63].

This disorder seems to be rare and few *TFR2* mutations have been reported: p.His33Asn, p.Glu60del, p.Arg105del, p.Met172Lys, p.Tyr250del, p.Gln317del, p.Arg396del, p.Ala444Thr, p.Arg455Gln, p.Arg481His, p.Leu490Arg, p.Val561del, p.Gln690Pro, and p.Gly792Arg. In both animal models and patients with *TFR2* related-HH decreased hepcidin levels were observed (Figure 1) [22,42,57,64–67].

### 2.4. SLC40A1

Type 4 HH (OMIM 606069) has an autosomal dominant pattern and it is caused by mutations in the *SLC40A1* gene. This rare disease can present peculiar clinical features such as high serum ferritin levels plus low or normal transferrin saturation values until the end stage of the disease. It may also be the presence of a mild iron-deficient anemia in the initial stage and a reduced tolerance to therapeutic phlebotomy [45,68,69].

*SLC40A1* (604353) gene, constituted by 8 exons, encodes a membrane transporter called ferroportin that modulates iron efflux [70]. *SLC40A1* mutations, such as p.His32Arg, p.Tyr64Asn, p.Val72Asp, p.Ala77Asp, p.Gly80Val, p.Arg88Thr, p.Asn144His, p.Asp157Gly, p.Asp157Asn, p.Val162del, p.Asn174Ile, p.Arg178Gly, p.Ile180Thr, p.Asp181Val, p.Gln182His, p.Asn185Asp, p.Gln248His, p.Gly267Asp, p.Gly323Val, p.Cys326Ser, p.Cys326Tyr, p.Gly330del, p.Ser338Arg, p.Arg489Ser, p.Gly490Asp, and p.Gly490Val were associated with type 4 HH. Two hypotheses have been proposed to account for this disease: the trapping of iron in macrophages that are unable to export iron and the failure to be degraded by interaction with hepcidin [22,42,69–74].

## 3. Biochemical Assays for Body Iron Store Analysis

The most common biochemical assays performed in laboratorial routine for iron overload analysis are serum iron, TIBC (total iron binding capacity), transferrin saturation (TS, which is a ratio between serum iron and TIBC expressed as percentage), and serum ferritin. Serum ferritin is a highly sensitive test for iron overload in HH, but it has low specificity, being also elevated in inflammatory process, diabetes, alcohol consumption, and liver damage.

Usually, TS values can be a helpful tool as a marker of iron overload. Some studies reported that TS values are usually higher than 50% in females and 60% in males with iron overload caused by genetic alterations [16,75–77]. In addition, a scale has been proposed by the Haute Autorité de Santé as clinical recommendations on the HH management: stage 0: without biochemical and clinical abnormalities; stage 1: increased TS (>45%), normal serum ferritin, and no clinical symptoms; stage 2: increased TS, increased serum ferritin (>200 μg/L in females and >300 μg/L in males), but no clinical symptoms; stage 3: abnormal biochemical values and initial clinical symptoms (fatigue, arthritis, impotence, skin hyperpigmentation); and stage 4: abnormal biochemical values, and clinical symptoms manifesting organ damage (cirrhosis, diabetes, hypogonadism, or cardiomyopathy) [76,78].

In this context, patients with suspect iron overload should primarily be evaluated through fasting TS and serum ferritin. *HFE* mutations molecular assay should be performed only in those with increased biochemical values [79].

## 4. Genetic Testing and Methodology

### 4.1. Genetic Testing

**Figure 2:**
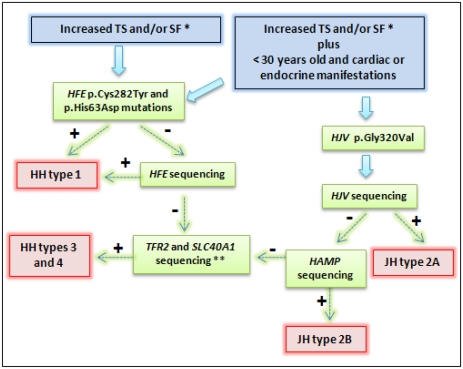
Representation of diagnostic strategy for patients suspected hereditary hemochromatosis (HH). * Recommendations report TS > 45%, SF > 200 μg/L in females and > 300 μg/L in males; or in advanced stages: TS > 50% in females and TS > 60% in males, in the absence of secondary causes [79,80]. ** Some patients with primary iron overload may not present mutation during this genetic approach. Very rare mutations in other genes can be involved [15,81]. Abbreviations: TS: transferrin saturation; SF: serum ferritin; JH: juveline hemochromatosis. + means positive result, and − means negative result.

*HFE* testing for the two main mutations (p.Cys282Tyr and p.His63Asp) should be performed in all patients with unexplained increased TS and/or serum ferritin values (Figure 2). In these cases, the molecular diagnostic of *HFE* related-HH is usually associated with the presence of the p.Cys282Tyr homozygosity and p.Cys282Tyr/p.His63Asp compound heterozygous genotypes. However, p.His63Asp homozygous and p.His63Asp/p.Ser65Cys compound heterozygous genotypes have been associated with HH phenotype [3,15,23].

In the absence of the mentioned *HFE* genotype combinations, other HH types could be considered. When there is genetic iron overload in a patient with less than 30 years and cardiac or endocrine manifestations, JH diagnostic is suggestive (Figure 2). Thus, the evaluation of the p.Gly320Val mutation in the *HJV* gene must be the molecular test of choice. According to several studies, this procedure would confirm the majority of JH cases [5,29]. Early diagnosis is paramount. If result is negative, sequencing should be performed to evaluate the *HJV* and *HAMP* genes (Figure 2). Our group reported a case with both clinical and molecular diagnostic of JH, and the use of deferasirox therapy adjunct to venesections during the initial treatment presented significant improvements as cardiomyopathy and liver disease were prevented, and endocrine functions were normalized [5].

Mutations in the *TFR2* and *SLC40A1* genes are rare compared with *HFE* mutations and they have also been reported in children, adolescents, and adults. These genes should be sequenced after negative results for other genes (Figure 2). Nowadays, the costs for sequencing have come down, especially if it evaluates the number of bases per dollar of the next generation sequencings. However, for the most part of clinical practice around the world, screening of *HFE*, *HJV*, *HAMP*, *TFR2* and *SLC40A1* through direct sequencing is not widely available. This approach is usually reserved for scientific studies and for very specific cases such as patients who are not responsive to treatment and had more severe complications due to iron overload. In addition and for the most part of cases, the treatment is not dependent on molecular diagnosis. Our group reported that direct sequencing does not significantly improve the diagnostic throughput as when it was compared to the sole *HFE* main mutations testing [15,19,79,80].

### 4.2. Methodology

Genotyping of *HFE* p.Cys282Tyr and p.His63Asp mutations is one of the most requested molecular assays in the laboratorial routine. The analysis of single nucleotide polymorphisms, for example *HFE* p.Cys282Tyr, *HFE* p.His63Asp, and *HJV* p.Gly320Val, can be performed by several available methods for genotyping (Figure 2), such as restriction fragment length polymorphisms (RFLP), allele-specific amplification analysis real time-polymerase, denaturing HPLC, sequencing strategies, TaqMan assay, multiplex amplification followed by reverse hybridization [79,82–84]. A high-resolutionmelting (HRM) assay was developed by our group for genotyping *HFE* p.Cys282Tyr and p.His63Asp mutations in a unique procedure being capable of ensuring the result in approximately 112 minutes and, with cost-effectiveness especially in a large-scale demand, compared to methods cited above. The advantages of genotyping with this procedure were the non-dependence on gel electrophoresis and on mutagenic reagents for visualization of fragments, and the reduction of the chances for contamination due to sample preparation compared to RFLP and sequencing strategies. There are disadvantages for the HRM method: interference from another genetic variant that may be present in the amplicon leading to misdiagnosis by altering the curve pattern of the target-mutation. The amplicon for the *HFE* p.Cys282Tyr mutation may present the following known genetic variants: p.Thr281Thr, p.Gln283Pro, p.Val284Met; and the amplicon for the *HFE* p.His63Asp mutation may present the p.Ser65Cys known genetic variants. Moreover, non-described mutations may also be present [83,85,86].

## 5. Conclusions

Advances in the understanding of HH have been obtained over the years: association of the *HFE* p.Cys282Tyr as the main mutation involved, genetic markers for juvenile hemochromatosis and several pathogenic mutations associated with non-*HFE* HH, hepcidin as an iron hormone, new techniques for the laboratorial evaluation, and increased knowledge about HH management. Nonetheless, there are still unclear points to be explored in the HH context: the exact role of the HFE protein, molecular pathways of the hepcidin synthesis, the identification of non-genetic factors that affect penetrance, more robust functional prediction tools, and protein functionality assays more informative and easier for the study of identified genetic alterations.

*HFE* testing for the two main mutations (p.Cys282Tyr and p.His63Asp) should be performed in all suspected patients with primary iron overload and unexplained increased TS and/or serum ferritin values. The evaluation of the *HJV* p.Gly320Val mutation must be the molecular test of choice in suspected patients with juvenile hemochromatosis.

In conclusion, hereditary hemochromatosis is an example that genetic testing can, in addition to performing the differential diagnostic with secondary iron overload, lead to more adequate and faster treatment.

## Figures and Tables

**Table 1:** Characteristics according to HH types.

HH types	Phenotype MIM number	Gene MIM number	Location	Inheritance	Gene product function	Main clinical manifestations
1	235200	*HFE*, 613609	6p21.3	AR	Involved in hepcidin synthesis via BMP6, interaction with TFR1.	Arthropathy, skin pigmentation, liver damage, diabetes, endocrine dysfunction, cardiomyopathy, hypogonadism.
2A	602390	*HJV*, 608374	1p21	AR	Involved in hepcidin synthesis, BMP co-receptor.	Types 2: earlier onset, <30 years old. Hypogonadism and cardiomyopathy more prevalent.
2B	613313	*HAMP*, 606464	19q13	AR	Downregulation of iron efflux from enterocytes.	
3	604250	*TFR2*, 604720	7q22	AR	Involved in hepcidin synthesis, interaction with transferrin.	As for type 1.
4	606069	*SLC40A1*, 604653	2q32	AD	Duodenal iron export.	Lower tolerance to phlebotomies and may have anemia.

MIM: Mendelian Inheritance in Man; TFR1: transferrin receptor 1, *HFE*: encodes HFE protein; *HJV*: encodes hemojuvelin; *HAMP*: encodes hepcidin; *TFR2*: encodes transferrin receptor 2; *SLC40A1*: encodes ferroportin; BMP6: bone morphogenetic protein 6, AR: autosomal recessive; AD: autosomal dominant.
